# Mitochondrial Uncoupler Prodrug of 2,4-Dinitrophenol, MP201, Prevents Neuronal Damage and Preserves Vision in Experimental Optic Neuritis

**DOI:** 10.1155/2017/7180632

**Published:** 2017-06-07

**Authors:** Reas S. Khan, Kimberly Dine, John G. Geisler, Kenneth S. Shindler

**Affiliations:** ^1^Scheie Eye Institute and FM Kirby Center for Molecular Ophthalmology, University of Pennsylvania, Philadelphia, PA 19104, USA; ^2^Mitochon Pharmaceuticals, Inc., 970 Cross Lane, Blue Bell, PA, USA

## Abstract

The ability of novel mitochondrial uncoupler prodrug of 2,4-dinitrophenol (DNP), MP201, to prevent neuronal damage and preserve visual function in an experimental autoimmune encephalomyelitis (EAE) model of optic neuritis was evaluated. Optic nerve inflammation, demyelination, and axonal loss are prominent features of optic neuritis, an inflammatory optic neuropathy often associated with the central nervous system demyelinating disease multiple sclerosis. Currently, optic neuritis is frequently treated with high-dose corticosteroids, but treatment fails to prevent permanent neuronal damage and associated vision changes that occur as optic neuritis resolves, thus suggesting that additional therapies are required. MP201 administered orally, once per day, attenuated visual dysfunction, preserved retinal ganglion cells (RGCs), and reduced RGC axonal loss and demyelination in the optic nerves of EAE mice, with limited effects on inflammation. The prominent mild mitochondrial uncoupling properties of MP201, with slow elimination of DNP, may contribute to the neuroprotective effect by modulating the entire mitochondria's physiology directly. Results suggest that MP201 is a potential novel treatment for optic neuritis.

## 1. Introduction

Optic neuritis is an inflammatory demyelinating disease of the optic nerve often associated with multiple sclerosis (MS) [1]. Optic neuritis may lead to complete or partial loss of vision in one or both eyes [2]. In 15%–20% of people who eventually develop MS, optic neuritis is their first symptom [3]. Current therapies used for optic neuritis, intravenous corticosteroids, show no benefit on final visual recovery, with up to 60% of patients failing to return to normal visual function, even though steroids have an effect in hastening acute visual recovery, decreasing inflammation, and reducing pain on eye movement associated with optic neuritis [1, 4].

In addition to inflammation and demyelination, optic neuritis is characterized by significant retinal ganglion cell (RGC) loss in the retina and axonal damage along the optic nerve, features that correspond to permanent visual loss [5, 6] that corticosteroids fail to prevent. Indeed, corticosteroids also show limited effect in preventing RGC axonal damage [7, 8]. Therefore, new therapies for the treatment of optic neuritis that specifically prevent RGC loss and preserve visual function are needed.

Mitochondrial dysfunction plays an important role in the neurodegeneration of optic neuritis and MS [9, 10]. Excessive reactive oxygen species (ROS) accumulation in the optic nerve is attributed to disease progression. Our studies have demonstrated compounds that modulate mitochondrial activity directly or indirectly attenuate ROS levels and disease progression by improved cellular function, improved biomarkers, and reduction in oxidative stress [11–14].

Mitochondrial uncoupling is the process in which protons (H^+^) generated indirectly via glycolysis of glucose and beta-oxidation of lipids, by generating NADH and FADH_2_ to be used in the electron transport system to generate a membrane potential by pumping protons out of the mitochondrial matrix, do not return through the ATPase channel to generate ATP once the membrane potential has been established [15]. This can happen from a naturally occurring phenomena coined “proton leak” in which ~25% of the body's potentially useful energy is dissipated as heat or via small molecule drugs called protonophores, ionophores, or uncouplers [16–18]. Protons leaking across the mitochondrial membrane are proposed as protective mechanism to minimize ROS generation during oxidative phosphorylation to slow aging [19]. Mitochondrial uncoupling also exists in hibernating animals and nonhibernating mammals such as humans [20, 21], but in low quantities [15, 22], in a specialized tissue called brown adipose tissue in which a protein channel called uncoupling protein 1 is highly expressed. In this case, protons travel into the mitochondria to generate heat [23]. There are other related uncoupling proteins (UCPs), which do not generate heat but are related to stress [24, 25]. Neurons express at least three UCP isoforms including the widely expressed UCP2 and the neuron specific UCP4 and UCP5, which play important roles in adaptive responses of neurons to oxidative stress. Mitochondrial uncoupling has been exhibited as a neuroprotective strategy in studies in which activity or overexpression of UCP2 exhibited improved functional recovery in models of epilepsy, ischemic stroke, Alzheimer's disease, and NMDA-induced retinal excitotoxicity [26–29]. Recent studies also have shown that pharmacological agents that induce mild mitochondrial uncoupling have tremendous therapeutic potential in a range of acute and chronic neurodegenerative conditions [25, 30–36].

MP201 is a prodrug of the mitochondrial uncoupler 2,4-dinitrophenol (DNP) that harnesses the power of the mitochondria by increasing energy expenditure that results in strengthening cellular survival [25], similar to the positive effects seen with fasting and exercise [37]. A recent study showed that mitochondrial uncoupling achieved by overexpression of UCP2 protected RGCs from glutamate excitotoxicity [29]. Thus, we hypothesized that MP201 may have similar neuroprotective properties that suppress RGC loss in optic neuritis and improve visual outcomes. The potential ability of MP201 to suppress optic neuritis and prevent RGC loss was examined in EAE mice.

## 2. Methods

### 2.1. Experimental Animals

Six-week old female C57/Bl6 mice were purchased from the Jackson Laboratory (Bar Harbor, ME, USA). Mice were housed at the University of Pennsylvania in accordance with university and National Institutes of Health guidelines. All procedures were approved by the Institutional Animal Care and Use Committee at the University of Pennsylvania.

### 2.2. Induction and Scoring of EAE

EAE was induced in C57/Bl6 mice according to a previously published protocol [11, 13, 38]. Briefly, 8-week old female mice were anesthetized with isoflurane and were given a total of 200 *μ*g of myelin oligodendrocyte glycoprotein (MOG) peptide (MOG_35–55_; Genscript, Piscataway, NJ, USA) emulsified in Complete Freund's Adjuvant (Difco, Detroit, MI, USA) containing 2.5 mg/ml *Mycobacterium tuberculosis* (Difco), administered via subcutaneous injections at two sites on the back. Control, sham-immunized mice were injected with an equal volume of phosphate buffered saline (PBS) and Complete Freund's Adjuvant. All animals received 200 ng pertussis toxin (List Biological, Campbell, CA, USA) in 0.1 ml PBS at 0 h and 48 h postimmunization, administered by intraperitoneal injection. EAE was scored using a previously published 5-point scale: no disease = 0; partial tail paralysis = 0.5; tail paralysis or waddling gait = 1.0; partial tail paralysis and waddling gait = 1.5; tail paralysis and waddling gait = 2.0; partial limb paralysis = 2.5; paralysis of one limb = 3.0; paralysis of one limb and partial paralysis of another = 3.5; paralysis of two limbs = 4.0; moribund state = 4.5; and death = 5.0 [11, 13, 38].

### 2.3. MP201 Treatment

MP201 (Mitochon Pharmaceuticals, Inc., Blue Bell, PA, USA) was dissolved in 1% DMSO, 40% polyethylene glycol 400 (PEG400), and 59% water and then diluted in PBS. EAE mice were treated by oral gavage (~300 *μ*L or 10 ml/kg) once daily with 16 or 80 mg/kg MP201 as indicated, starting from day 15 postimmunization until sacrifice (day 42). MP201 is a prodrug of DNP with ~10x lower exposure to DNP at the same doses. Prior studies have shown that the therapeutic window between 0.5, 1, and 5 mg/kg of DNP is neuroprotective [25]. To dose at equivalent exposures of 1 and 5 mg/kg of DNP with MP201, there is a factor of 10x, plus the extra molecular weight (1.6x) of the prodrug, thus 16 and 80 mpk provide equivalent exposures. Control, non-EAE mice, and sham-treated EAE mice were treated with an equal volume (~300 *μ*L or 10 ml/kg) of PBS.

### 2.4. Measurement of Optokinetic Responses (OKR)

Optokinetic responses (OKR) were used to assess visual function in control and EAE mice treated with or without MP201. OKR were measured as the highest spatial frequency at which mice track a 100% contrast grating projected at varying spatial frequencies using OptoMetry software and apparatus (Cerebral Mechanics Inc., Medicine Hat, AB, Canada), as in prior studies [11, 38]. Data are reported as cyc/deg.

### 2.5. Quantification of Retinal Ganglion Cell Survival

RGCs were immunolabeled in flat-mounted retinas and counted as described previously [11, 38]. Briefly, retinas isolated from mice following sacrifice at day 42 were prepared as flattened whole mounts. Retinas were then permeabilized in 0.5% Triton X-100 in PBS by freezing for 15 min at −70°C followed by washing in PBS containing 0.5% Triton X-100. Specimens were then incubated overnight at 4°C with goat-anti-Brn3a antibody (RGC marker) (Santa Cruz Biotechnology, Dallas, TX, USA) diluted 1 : 100 in PBS containing 2% bovine serum albumin and 2% Triton X-100 (blocking buffer). The retinas were washed three times in PBS, incubated for 2 hours at room temperature with Alexa Fluor 488-conjugated anti-goat secondary antibody (Thermo Fisher Scientific, Waltham, MA, USA), diluted 1 : 500 in blocking buffer, washed in PBS 3 to 4 times, and mounted vitreous side up on slides in fluorescent anti-fading solution. RGCs were photographed at 40x magnification in 12 standard fields: 1/6, 3/6, and 5/6 of the retinal radius from the center of the retina in each quadrant, and counted by a masked investigator using image analysis software (Image-Pro Plus 5.0; Media Cybernetics, Silver Spring, MD).

### 2.6. Quantification of RGC Axon Staining

Neurofilament staining in optic nerve sections was done to quantify the area of intact RGC axons using a previously published protocol [11, 38]. Briefly, optic nerves were isolated following sacrifice after 42 days, fixed in 4% paraformaldehyde, and embedded in paraffin. 5 μm longitudinal paraffin sections of the optic nerve were deparaffinized, rehydrated, and then permeabilized with 0.5% tween-20 in PBS. Blocking reagent (Vector Laboratories, Burlingame, CA, USA) was used to reduce nonspecific binding. Specimens were then incubated in rabbit anti-neurofilament antibody 1 : 100 (Abcam, Cambridge, MA, USA) at 4°C overnight and then washed three times with PBS and incubated with anti-rabbit secondary antibody (Vectastain Elite ABC Rabbit kit) for 30 min at 37°C. Avidin/Biotin Complex detection was performed using the Vectastain Elite ABC kit and DAB (diaminobenzidine) substrate kit (Vector Laboratories) according to the manufacturer's instructions. Photographs of three fields/nerve (one each at the distal, central, and proximal regions of the longitudinal optic nerve section) at 20x magnification were taken by a blinded investigator. Neurofilament staining was quantified by calculating the optical density using ImageJ software (http://nih.gov).

### 2.7. Quantification of Demyelination in the Optic Nerve

Luxol fast blue (LFB) staining was used to evaluate demyelination in the optic nerves. Optic nerve sections were stained with LFB as in prior studies [11, 38], and the entire length of each optic nerve section was examined by light microscopy by a blinded investigator. Demyelination in optic nerves was quantified on a 0–3 point relative scale: 0 = no demyelination; 1 = scattered foci of demyelination; 2 = prominent foci of demyelination; and 3 = large (confluent) areas of demyelination.

### 2.8. Quantification of Inflammation in the Optic Nerve

Hematoxylin and Eosin (H&E) staining was used to evaluate inflammation in the optic nerves. Optic nerve sections were stained with H&E as in prior studies [11, 38], and the entire length of each optic nerve section was examined by light microscopy by a blinded investigator. Presence of inflammatory cell infiltration in the optic nerves was assessed according to a 0–4 point scale: 0 = no infiltration; 1 = mild cellular infiltration of the optic nerve or optic nerve sheath; 2 = moderate infiltration; 3 = severe infiltration; and 4 = massive infiltration.

### 2.9. Statistics

Data are expressed as means ± SEM. Differences in OKR across time were compared by ANOVA of repeated measures using GraphPad Prism 5.0 (GraphPad Software, San Diego, CA, USA). Differences in RGC numbers, RGC axon staining, inflammation, and demyelination were compared using one-way ANOVA followed by Student-Newman-Keuls test using GraphPad Prism 5.0. Differences were considered statistically significant at *p* < 0.05. For all experiments, each eye was used as an independent data point similar to prior studies [11–14], based on previous studies showing that optic neuritis can occur bilaterally, or unilaterally in either eye, and thus can occur as an independent event.

## 3. Results

### 3.1. MP201 Treatment Preserves Visual Function

**Figure 1:**
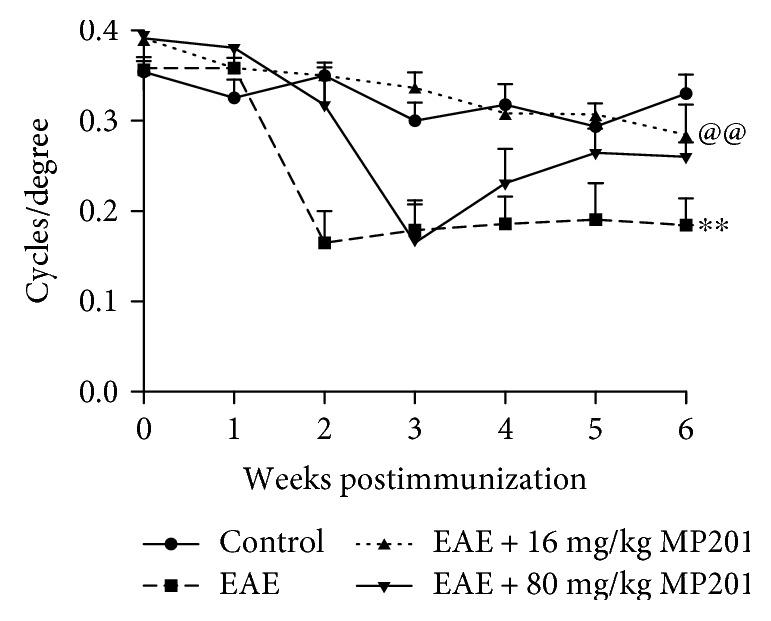
MP201 preserves RGC function. Visual function, measured by OKR responses, shows significant (^∗∗^*p* < 0.01) decreases in eyes of EAE mice (*N* = 10 eyes) compared to control mouse eyes (*N* = 10) by 6 weeks after induction of EAE. Daily oral treatment of 16 mg/kg MP201 (*N* = 10) from days 15 to 42 postimmunization leads to significantly (@@*p* < 0.01 versus EAE) improved OKR responses in EAE mice. Mice receiving 80 mg/kg MP201 daily (*N* = 10) show initial vision loss by week 3 that then reverses with improved OKR score in subsequent weeks.

Eight-week old female C57BL/6J mice were immunized with MOG_35–55_ peptide to induce EAE, or sham-immunized with PBS as controls. Mice were sham-treated with PBS, or treated with 16 or 80 mg/kg MP201, daily by oral gavage beginning after onset of optic neuritis on day 15 postimmunization. OKR was measured every week until sacrifice at day 42. By day 42 postimmunization, OKR responses were significantly decreased in the eyes of PBS-treated EAE mice as compared to both control mice and EAE mice treated with either 16 or 80 mg/kg MP201 (Figure 1). EAE mice that received 80 mg/kg MP201 daily showed an initial vision loss at week 3 that reversed and improved significantly at later time points. EAE mice that received 16 mg/kg MP201 daily showed a significant improvement in OKR scores at all time points compared to PBS-treated EAE mice (*p* < 0.01).

### 3.2. MP201 Treatment Reduces RGC Loss in the Retina

**Figure 2:**
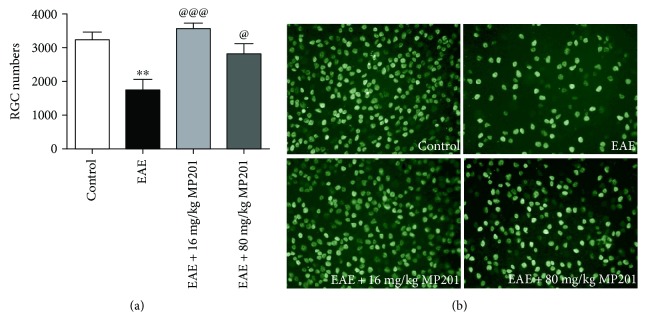
MP201 treatment attenuates RGC loss. Neuroprotective effects of MP201 were evaluated by counting RGCs immunolabelled with Brn3a antibody in 12 standardized fields, three from each quadrant of the retina. (a) RGC loss in eyes of EAE mice (^∗∗^*p* < 0.01 versus control, *N* = 10 eyes) is reduced by 16 mg/kg MP201 treatment from days 15 to 42 (@@@*p* < 0.001 versus EAE, *N* = 10). 80 mg/kg MP201 also induces a significant (@*p* < 0.05 versus EAE, *N* = 10) improvement in RGC numbers. (b) Representative images show RGCs in one field of retina from each group (original magnification ×20).

Previous studies demonstrate that MOG-induced EAE mice develop significant RGC loss 30 to 40 days postimmunization and that this model can be used to evaluate the neuroprotective potential of therapies to prevent RGC loss [11, 13, 38]. To examine potential neuroprotective effects of MP201, PBS- and MP201-treated mice were sacrificed 42 days postimmunization, and RGCs were counted in isolated retinas. RGC numbers in eyes from PBS-treated EAE mice showed a significant decrease (*p* < 0.01) compared to control mice (Figure 2). Daily treatment on days 15–42 postimmunization with either 16 mg/kg MP201 (*p* < 0.001) or 80 mg/kg MP201 (*p* < 0.05) led to a significant attenuation of RGC loss compared to PBS-treated EAE mice.

### 3.3. MP201 Treatment Reduces RGC Axonal Loss in Optic Nerve

**Figure 3:**
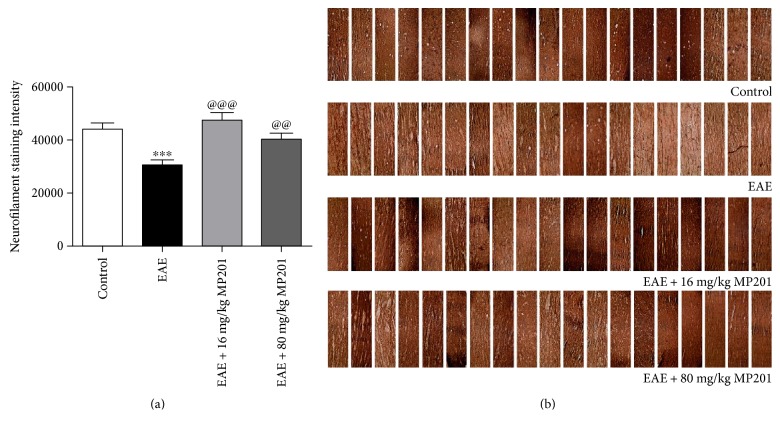
MP201 treatment reduces axonal loss in optic neuritis. Neurofilament staining was used to evaluate axonal loss in sections of optic nerves isolated at day 42 postimmunization. (a) The optical density of neurofilament staining calculated from three equal-sized fields from each optic nerve shows a significant decrease (^∗∗∗^*p* < 0.001) in optic nerves (*N* = 10 nerves) from EAE mice compared to optic nerves (*N* = 10) from control mice. Treatment with 16 mg/kg (@@@*p* < 0.001, *N* = 10) or 80 mg/kg (@@*p* < 0.01, *N* = 10) MP201 induces a significant increase in neurofilament staining compared to optic nerves from PBS-treated EAE mice. (b) A series of photographs of axon staining from multiple optic nerve sections shows the normal degree of variability of neurofilament staining in optic nerves of control mice and EAE mice treated with 16 mg/kg or 80 mg/kg MP201 and shows more patchy loss of neurofilament staining in optic nerves from PBS-treated EAE mice.

Neurofilament staining was used to quantify axon density in PBS- and MP201-treated EAE mice as in prior studies [11, 38]. Significant (*p* < 0.001) reduction in axonal staining occurred in optic nerves from PBS-treated EAE mice as compared to optic nerves from control mice (Figure 3()), similar to prior studies [11, 38]. Treatment with 16 mg/kg of MP201 showed a significant (*p* < 0.001) attenuation of optic nerve axonal loss compared to PBS-treated EAE mice, and treatment with 80 mg/kg also resulted in a significant (*p* < 0.01) reduction in axonal loss.

### 3.4. MP201 Treatment Reduces Demyelination in Optic Nerve

**Figure 4:**
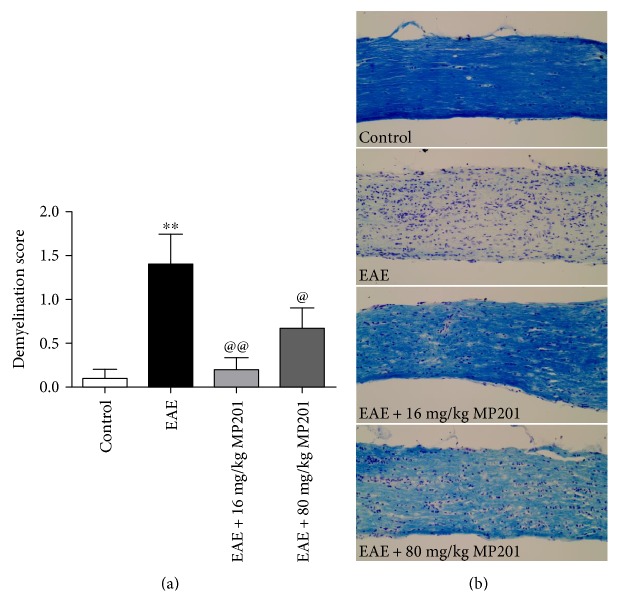
MP201 treatment attenuates demyelination in optic nerves during EAE. To examine whether MP201 treatment prevents demyelination, optic nerves were isolated from mice 42 days postimmunization and were stained with LFB. (a) LFB-stained optic nerve longitudinal sections were examined by a blinded investigator, and demyelination was quantified on a 0–3 point scale. Optic nerves (*N* = 10 nerves) from EAE mice had a significantly (^∗∗^*p* < 0.01) higher demyelination score compared to optic nerves (*N* = 10) from control mice, and treatment with 16 mg/kg (@@*p* < 0.01 versus EAE, *N* = 10) or 80 mg/kg (@*p* < 0.05 versus EAE, *N* = 10) MP201 leads to a significant decrease in demyelination scores. (b) A representative image of one optic nerve from a PBS-treated EAE mouse shows less LFB (blue) staining than an optic nerve from a control mouse, as well as optic nerves of EAE mice treated with either 16 mg/kg or 80 mg/kg MP201 (original magnification ×20).

Inflammatory demyelination of RGC axons leading to poor nerve conduction is believed to be a prominent pathophysiology of optic neuritis [39]. To examine whether MP201 can block demyelination, optic nerves from control, PBS-treated EAE, and MP201-treated EAE mice were stained with LFB and evaluated for demyelination. PBS-treated EAE mice showed a significant increase in demyelination score compared to control mice (*p* < 0.01) (Figure 4), consistent with previous studies [11, 38]. Daily treatment with 16 mg/kg (*p* < 0.01) and 80 mg/kg (*p* < 0.05) resulted in a significant suppression in the degree of demyelination in EAE optic nerves.

### 3.5. MP201 Treatment Does Not Affect Optic Nerve Inflammation

**Figure 5:**
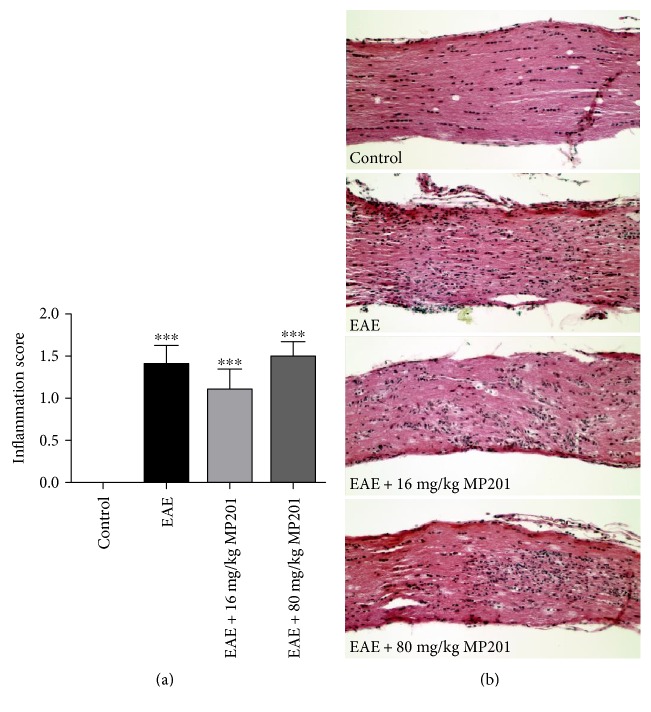
MP201 treatment does not suppress inflammation in optic nerves during EAE. To examine whether MP201 treatment prevents inflammation, optic nerves were isolated from mice 42 days postimmunization and stained with H&E to examine levels of cellular infiltration. (a) H&E-stained optic nerve longitudinal sections were examined by a blinded investigator, and inflammation was quantified on a 0–4 point scale. Optic nerves (*N* = 10 nerves) from EAE mice showed significantly (^∗∗∗^*p* < 0.001) higher inflammation scores compared to optic nerves (*N* = 10) from control mice, and treatment with 16 mg/kg (^∗∗∗^*p* < 0.001 versus control, *N* = 10) or 80 mg/kg (^∗∗∗^*p* < 0.001 versus control, *N* = 10) showed no change in inflammation score compared to EAE. (b) A representative image of one optic nerve from a PBS-, 16 mg/kg MP201-, and 80 mg/kg MP201-treated EAE mouse each show increased numbers of cells, representative of inflammatory cell infiltration, as compared to an optic nerve from a control mouse (original magnification ×20).

Optic nerves of EAE mice treated with PBS or with MP201 were stained with H&E to evaluate inflammation. Significant (*p* < 0.001) inflammatory cell infiltration was detected in optic nerves from PBS-treated EAE mice as compared to control mouse optic nerves that demonstrated normal histology without inflammation (Figure 5). Optic nerves of EAE mice treated daily with both 16 mg/kg and 80 mg/kg MP201 also showed significant inflammatory cell infiltration compared to control mouse optic nerves, with no statistical difference compared to optic nerves from PBS-treated EAE mice.

## 4. Discussion

Results indicate that oral administration of MP201 provides significant neuroprotective benefits in an experimental model of optic neuritis. MP201 treatment led to a significant reduction in the loss of RGCs and their axons and attenuated demyelination along optic nerves. In addition to these structural effects, MP201 also helped preserve OKR responses. OKR is an objective means of detecting visual activity in mouse [40]. The optokinetic system plays an essential role in stabilizing the visual image on the retina by producing compensatory eye movement in the direction of visual motion and can be used as a marker of RGC function [41]. Previous studies have shown that OKR responses decrease in EAE mice and some treatments that prevent RGC loss preserve OKR responses [11, 38], consistent with the current results. Thus, MP201 promotes improvement in both structural and functional outcomes in experimental optic neuritis.

Mitochondrial oxidative stress and loss of mitochondrial membrane potential are classically believed to be major mediators of many neurodegenerative diseases [42, 43], and accumulating evidence suggests that oxidative stress plays a major role specifically in the pathogenesis of MS and optic neuritis [44, 45]. Indeed, various treatment strategies that reduce oxidative stress show promising neuroprotective effects in models of optic neuritis and other optic neuropathies. Previous studies have shown that increasing mitochondrial defenses against accumulating superoxide protect RGCs and their axons [46]. In addition, viral-mediated gene delivery of antioxidant genes MnSOD (manganese superoxide dismutase) and catalase are effective in suppressing not only myelin loss in the optic nerve but also mitochondrial vacuolization, optic nerve head swelling, and dissolution of cristae in optic nerve axons [46, 47]. Our previous studies show significant RGC neuroprotective effects mediated by compounds that activate SIRT1 (Sirtuin 1, silent mating type information regulation 2 homolog) in both EAE- and mouse hepatitis virus-induced optic neuritis [11, 13, 14, 48] and similar neuroprotective effects of SIRT1 overexpression in a traumatic optic nerve injury model [49]. While these studies show SIRT1 activation significantly attenuates RGC damage by reducing oxidative stress via deacetylation of PGC1*α* (peroxisome proliferator-activated receptor gamma coactivator 1-alpha) and subsequent increase in mitochondrial biogenesis, effects on visual function are only transient. Thus, previous studies show that mitochondrial biogenesis and reduction of oxidative stress are important targets for neuroprotective therapies in optic neuritis, but the significant improvement in visual function following MP201 treatment in the current studies suggests that this mitochondrial uncoupling therapy provides stronger neuroprotective effects than other antioxidant treatment strategies.

Mild mitochondrial uncoupling is proposed to be one of the central mechanisms through which oxidant production is controlled in mitochondria [50, 51]. Overexpression of UCPs in neuronal cells results in better preservation of cellular ATP levels and lower oxidative stress with normal mitochondrial morphology in vitro [52, 53]. UCPs promote neuroprotective effects by diminishing oxidative stress in models of Parkinson disease [54], focal cerebral ischemia [30] seizures [26, 55], and brain trauma [30, 56], and UCP2 itself exerts protective effects in EAE [57]. In addition to UCPs, pharmacological agents that induce mitochondrial uncoupling are effective therapeutic tools for preventing neurodegeneration in a wide range of neurodegenerative diseases [30–33]. The compound used in present study, MP201, is a prodrug of DNP, the most widely studied and consistently effective mitochondrial uncoupling agent in experimental models of neurodegeneration [25, 32, 58, 59]. MP201 has a linker on the hydroxyl group that caps the oxygen, but once it crosses into portal vein, enzymes cleave the linker and the oxygen group gets protonated to the active form of DNP [60]. DNP can prevent calcium accumulation and related ROS generation to promote neuroprotective effects [32, 36, 61] and stimulates cAMP (cyclic adenosine monophosphate) production, tau expression (nonphosphorylated), and neurite outgrowth in cultured neuronal cells at low concentrations [62]. Low doses of DNP ameliorate learning and memory deficits in a mouse model of Alzheimer's disease [25] and protect neurons against dysfunction and degeneration in experimental models of ischemic stroke [58], traumatic brain injury [32], Huntington disease [36], and peripheral nerve injury [59]. DNP at low doses appears to provide a benefit in models of known and unknown genetic causes, including epilepsy [25]. Collectively, data suggest that DNP may be a broad spectrum treatment for a host of indications associated with mitochondrial dysfunction, without necessarily mitochondrial mutations. Interestingly, a recent study shows that enhanced mitochondrial uncoupling by overexpression of UCP2 decreases apoptosis in RGCs and protects against the toxic effects of glutamate agonists by regulating ROS production [29]. Overall, previous studies support the concept that the mitochondrial uncoupling property of DNP likely contributes to its neuroprotective actions. Therefore, preservation of RGCs and improved visual function mediated by MP201 in the current studies may be related to its mitochondrial uncoupling properties after conversion to DNP.

DNP was used as a medication in the 1930's for weight loss, but a host of adverse side effects prompted a ban on its use as a prescription drug [63–65]. For our present study, we used MP201, a prodrug of DNP that may reduce the risk of overdose and abuse of DNP, and we used MP201 doses that generate DNP at doses ~10 to 50 times lower than the dose used in the past for weight loss [25], suggesting that this hormetic-like therapy may be much better tolerated than past treatment with DNP directly. Other potential beneficial features of MP201 are its ability to suppress the Cmax of DNP 10-fold, triple its elimination time, and apparent ability to improve its pharmacology, perhaps due to its trickle-like systemic delivery [60]. The potential safety of DNP at lower doses distributed over time is supported by recent studies showing no evidence of toxic effects during chronic administration of controlled-release of DNP [66]. Previous literature supports this idea, as chronic low dose treatment of DNP in drinking water (mimicking controlled release) increased longevity with low levels of oxidative proteins and DNA damage in mice [67]. One eye-related potential side effect of DNP is cataracts, which were reported in some patients treated with high doses of DNP [68], but the result was not replicated in follow-up experiments [69]. Given that the significant neuroprotective effects found in the current studies were induced by essentially a sustained release prodrug formulation of DNP requiring much lower doses than those found to be toxic in the past, this novel prodrug therapy warrants further investigation as a potential neuroprotective therapy.

Interestingly, the higher dose of MP201, 80 mg/kg, examined here did not lead to improved neuroprotective effects as compared to the lower dose, 16 mg/kg. This may suggest that effects of MP201 reach a homeostatic or hormetic level at low doses that lessens at higher doses. This is consistent with previous studies that found low sustained levels of DNP administration ameliorate neurological disease processes and improve functional outcomes, without reducing body weight as seen at much higher doses [25, 58, 61, 70].

## 5. Conclusion

The current data demonstrate potential neuroprotective effects of MP201, a prodrug of the mitochondrial uncoupling agent DNP, in experimental optic neuritis. MP201 represents a promising new potential therapy for use in optic neuritis and other optic neuropathies which warrants further investigation.

## Abbreviations

DNP: 2,4-Dinitrophenol
RGC: Retinal ganglion cells
EAE: Experimental autoimmune encephalomyelitis
ROS: Reactive oxygen species
UCPs: Uncoupling proteins
MOG: Myelin oligodendrocyte glycoprotein
OKR: Optokinetic responses
H&E: Hematoxylin and Eosin
LFB: Luxol fast blue.
